# A Systematic Review and Meta-Analysis of the Effective Contribution of Indian Periodontists to Global Periodontal Research Advancement

**DOI:** 10.7759/cureus.33056

**Published:** 2022-12-28

**Authors:** Hiroj Bagde, Ashwini Dhopte

**Affiliations:** 1 Department of Periodontology, Rama Dental College and Research Centre, Kanpur, IND; 2 Department of Oral Medicine and Radiology, Rama Dental College and Research Centre, Kanpur, IND

**Keywords:** periodontitis, periodontics, periodontia, periodontal, gingival, gingiva, dental implant, dental

## Abstract

Developed nations put a lot of emphasis on scientific research and produce an enormous volume of avant-garde papers in impactful journals. These publications could serve as the foundation for different policies or other designs. The researchers in this study aimed to use a quantitative bibliometric strategy to analyze the development of Indian periodontists' publishing patterns in the PubMed database up to October 31, 2022. Publications that could be accessed through the PubMed database as of the end of October 2022 were included in the bibliometric study. By using certain search terms on the PubMed search engine, studies were found. Dental, periodontal, gingival, gingivitis, periodontal, periodontitis, and dental implants were among the terms used to find this article. To evaluate articles that are specifically about India, a parallel search was conducted with the following phrases together with "India." Selected parameters were examined in all papers, whether they had or lacked abstracts. Seven studies were selected which were in accordance with the inclusion and exclusion criteria of this study. According to the keyword search, India contributes an average of 5.65% of each keyword category to the PubMed database, since the total number of search results on PubMed for the seven keywords we entered was 1,037,584, and the same keywords when searched by adding the keyword "India" to the keyword generated a total of 58,624. Since the beginning of the last decade, India has recorded tremendous growth in all spheres of scientific literature publication, and the field of periodontics is no exception. Through the PubMed database, Indian periodontists have made a significant contribution to world literature, especially over the past 10 years, with the number of publications increasing nearly exponentially with each passing year.

## Introduction and background

Dental science, also known as dentistry, is a subspecialty of medicine that focuses on the diagnosis, treatment, and management of oral health issues, such as tooth misalignment and mouth deformities [1]. Indian dental researchers are putting a lot of effort into examining patients, diagnosing, and treating cavities in the mouth and related ailments in their offices and hospitals. Doctors of dental surgery and dentists who teach at dental schools collaborate on research and write articles about their findings side by side. Endodontics, orthodontics, periodontics, prosthodontics, pedodontics, oral and maxillofacial surgery, restorative dentistry, oral pathology, oral radiology, oral medicine, and many others are all important branches of dental science. In that year, the American Diary of Dental Science, which is considered to be the first dental journal, began its distribution [2]. The primary source of information used to refresh dentists' knowledge is journals. The Dentists Act of 1948 regulates dental education and practice in India through the Dental Council of India [3].

The goal of scientific communication is to disseminate research findings to the entire scientific community as well as to one's peers. One of the essential elements in evaluating the output of research in an academic environment is research articles. It transmits data to upcoming researchers with the intention of disseminating and reusing it for societal advancement. Contributing to existing knowledge or facts is what research is all about. Through indicators such as publication counts, citation counts, keyword analysis, co-citation analysis, and other metrics, scientometrics assesses the research contribution of a field. According to Subramanyam [4], the evaluation of collaboration should highlight a holistic approach. He also claimed that the complexity of human interaction makes it challenging to quantify the nature of research collaboration. The collection of techniques used for quantitatively assessing academic literature is known as bibliometrics.

The development of Indian dental science can be evaluated with the aid of high-caliber research articles. Some of the major bibliometric measures used to gauge research production include the number of publications, the h-index, citation counts, and impact factors (IFs) of journals. It may be useful to learn about leading dental institutions' and dentists' international partnerships for the advancement of dental science through research collaboration in the field. There are many different forms of research investigations in periodontics scientific publications. These studies contribute to the discovery of new therapies and the appropriate application of existing treatment modalities through the provision of evidence-based findings. Bibliometrics refers to the quantitative information obtained from these scientific findings using a core methodological approach by the authors and their collaborators. Thus, the term "bibliometric research" refers to a quantitative and statistical method that focuses on goals and observable indications of academic activity, namely, publications and citations [5]. Additionally, it examines how the editorial standards of scientific journals affect scientific communication, offering room for the research's quality to advance. This is done by counting the number of documents a nation or researcher has published, as well as the number of citations each article has received. The citations aid in understanding the article's significance and impact on the field of clinical practice today [6].

According to the PubMed database, the first study on dental/oral sciences in India was published in 1925 [7]. Then, in 1946, Shourie's essay was the first by an Indian author to be published in the PubMed database [8]. It was not until 1960 that Muller and Zander published cementum from Indian teeth with periodontal disease in the PubMed database [9]. An article by Chawla and Mehta, the first major work by an Indian periodontist in a public distribution from an Indian foundation, was added to the PubMed database in 1960 [10]. Instead, in 1965, Rao, Shourie, and Shankwalkar wrote a book that they distributed internationally and is considered the standard work for Indian periodontists [11]. The USA provided training and education for the first periodontists in India. Back in India, they established dental schools, departments of periodontology, and specialized clinics. They launched research projects and publications in addition to starting to monitor the development of periodontics in India. Several researchers have undertaken bibliometric evaluations of the output of research in various world locations and with regard to a particular topic [12-14]. The differing approaches employed, however, limit comparisons between research outcomes in other scientific fields [15].

The PubMed/MEDLINE database is one of the biggest libraries dedicated to medical literature. The world's population may always have access to this excellent resource. These databases, which are virtually updated daily, were developed and are maintained by the United States National Center for Biotechnological Information and the National Institutes of Health. Furthermore, the quality of individual articles and academics is often evaluated according to the IFs of the journals in which they were published. The SCImago Journal Rank (SJR) indicator, a journal metric system, calculates the SJR value, or the citation weight, of that specific journal for the given year, by averaging the citations over the previous three years [16]. Higher SJR levels indicate a high journal IF [17].

The purpose of this research was to analyze the scientometric data of a selection of papers documenting Indian periodontists and their impact on the field. It is important to evaluate India's contribution to scientometrics and to understand the status and significant advancements in periodontics relative to other nations.

## Review

Materials and methods

Protocol Employed 

The Preferred Reporting Items for Systematic Review and Meta-Analysis (PRISMA) protocol and guidelines from the Cochrane group and the book "Orderly Reviews in Health Care: Meta Examination" were followed for conducting this systematic review [18].

Search Strategy 

For the bibliometric analysis, only publications that were accessible through the PubMed database as of the end of October 2022 were included. The data search was conducted on October 31, 2022, at 11 a.m. Indian Standard Time (IST) and continued until 12 p.m. IST, utilizing the most recent version of the Google Chrome internet browser. The terms dental, periodontal, gingiva, gingival, periodontia, periodontitis, periodontology, and dental implant were used to conduct individual searches. The same search was conducted again, but for each term, the word "India" was added (e.g., "dental," "India," "oral," etc.). The keywords were entered exactly where they were supposed to be on the PubMed website homepage.

Study Selection 

All of the keyword categories yielded all of the article abstracts. Then, for each category, only publications that connected to periodontology were manually looked for and chosen. Comparing between categories, overlapping articles were eliminated. Separate searches were conducted for Dental Dialogue (DD), the Journal of the Indian Dental Association (JIDA), and the Journal of the Indian Society of Periodontology (JISP). These Indian journals were looked up individually because they were not reliable in their publications and only occasionally appeared in the PubMed database (JISP was added to PubMed in January 2008 and later).

Inclusion Criterion

Speaking about the inclusion criteria used for our study, only papers that were still accessible in the PubMed database as of October 31, 2022 were chosen. Additionally, only publications by periodontists of Indian origin that came from India were taken into consideration for the purposes of our analysis. In other words, papers that appear in journals that are indexed by PubMed, of Indian origin, were taken into consideration.

Exclusion Criterion

The article did not contain an introduction, editorials, messages, letters to the editor, obituaries, or organizational communications. Only newly authored works, such as articles, reviews, or case studies/series, were included. Studies with specific flaws, such as incorrectly citing the name of the institution or department, omitting to cite the state of origin, or lacking abstracts, were not taken into consideration for our analysis. Articles that did not identify the authors, the name of the institute, the departmental affiliation, or any other information outside the topic of the article were also disqualified.

Statistical Analysis

**Figure 1:**
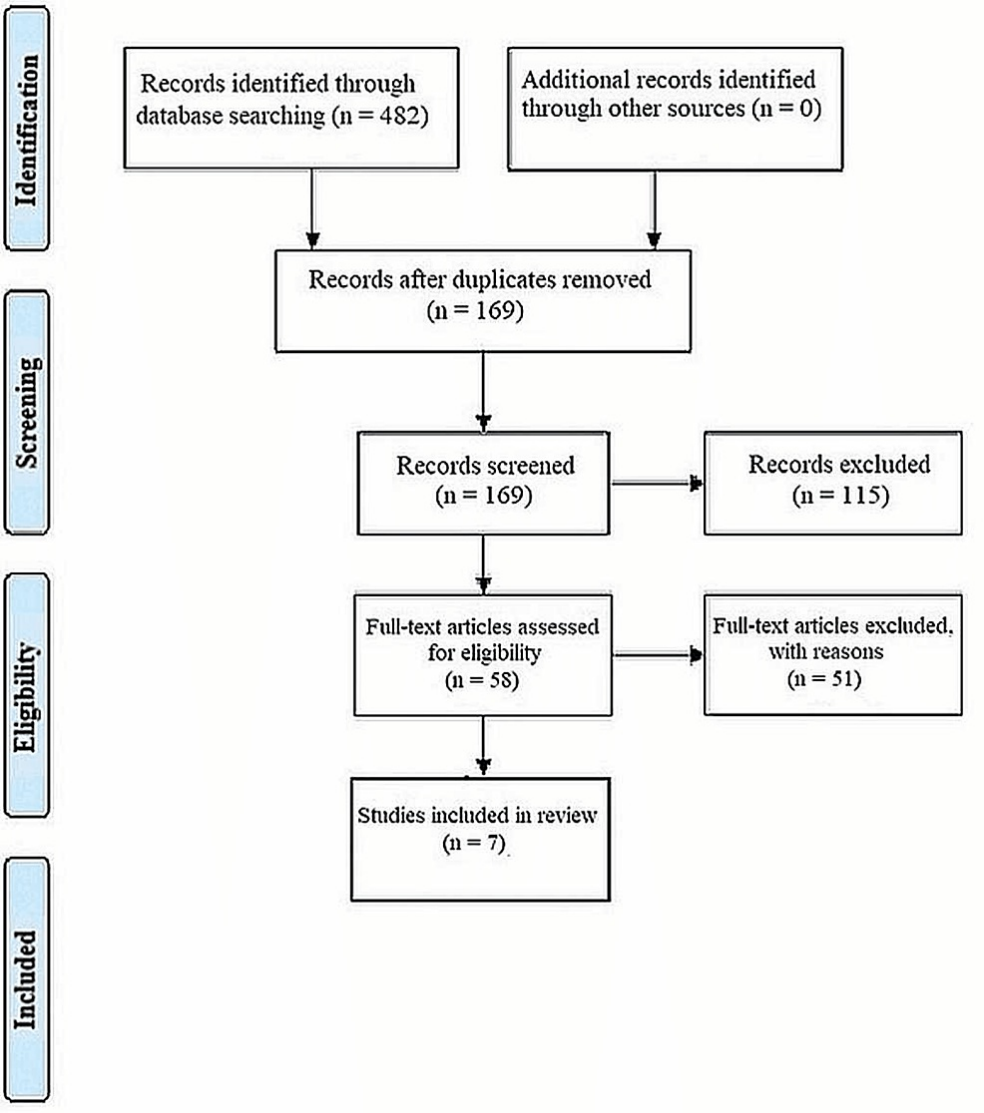
PRISMA protocol for the articles included PRISMA: Preferred Reporting Items for Systematic Review and Meta-Analysis

The results of the meta-analysis, in the form of forest plots depicting all the studies taken up in this systematic review, were generated using the RevMan 5 software (Cochrane Interactive Learning, England and Wales). Data on the variables analyzed and different aspects of the investigations selected for our systematic review were entered into the software, and the forest plots representing the risk ratio, odds ratio, and risk difference were obtained as part of the meta-analysis for our review (Figure 1).

Results

As can be seen in Table 1, the number of overall total search results on PubMed for the seven keywords that we entered was 1,037,584, and the same keywords, when searched by adding ‘India’ to the keyword, generated a total of 58,624, which means according to our keyword research, India contributes an average of 5.65% of each keyword category to the PubMed database.

**Table 1:** PubMed search results with the keywords with and without the keyword "India"

Search keyword	Number of results obtained (overall)	Search keyword with "India" added	Number of results obtained (overall)	Percentage of keyword search results with "India"
"Dental"	636,263	"Dental", "India"	36,177	5.68%
"Gingiva", "Gingival"	24,836	"Gingiva", "Gingival", "India"	1,168	4.7%
"Periodontology"	68,215	"Periodontology", "India"	5,811	8.52%
"Periodontics"	120,716	"Periodontics", "India"	6,536	5.41%
"Periodontia"	11,298	"Periodontia", "India"	348	3.08%
"Periodontitis"	120,716	"Periodontitis", "India"	6,536	5.41%
"Dental implant"	55,540	"Dental implant", "India"	2,048	3.69%

The science citation index (SCI) expanded (now owned by Clarivate Plc., London, United Kingdom) and JCR Science edition 2021- Impact factor (updated June 2022) were used to evaluate the IFs for the journals listed in the studies selected for this systematic review [19].

From its first publication in 1960 until its most recent in 2004, there were not many more. The percentage of publications published declined to 29.32% between 1960 and 2005. The total number of publications has almost steadily risen from 2007 to 2021. In 2021, there were 604 books released, an all-time high.

The results of the systematic review have been tabulated in Table 2 presented below, with the details of the six studies that were selected for the review presented in the table.

**Table 2:** Descriptions and outcomes of the selected studies in this systematic review and meta-analysis

Author and year of study	Study design	Study description	Study inference
Baghele et al., 2012 [20]	Bibliometric assessment	The purpose of this study was to evaluate the trends of publications by Indian periodontists in the PubMed database up to the first of March, 2012, using a quantitative bibliometric approach. By using certain search terms on the PubMed search engine, studies were found. To evaluate publications that were specifically on India, a parallel keyword search using India was also conducted. Selected parameters were examined in all papers, whether they had or lacked abstracts. Specifics about the journal's name, authorship, publication date, publisher, journal's reach inside the state, study's methodology, etc. were all determined through analysis.	Until the first of March 2012, Indian dental and periodontal literature had contributed roughly 1.45% to the PubMed database. 764 publications written by Indian periodontists were published in 107 journals commencing in 1960. There were 510 (66.75%) original publications published as opposed to 127 (16.62%) review articles and case reports/case series combined. An Indian periodontist contributed 0.53 articles on average to the PubMed database.
Christina et al., 2022 [21]	Bibliometric assessment	Using the Boolean search string (TS=periodont* OR gingiv*) AND (Year=1989 to 2018), the data was obtained from the WoS (Web of Science) platform. There were 69952 bibliographic records retrieved in total. The information was once more divided into three time periods: 1989 to 1998, 1999 to 2008, and 2009 to 2018. Similar to this, information about India was sifted and retrieved for three decades.	According to the data collected, no periodontics-related paper from India has appeared in this journal between 1989 and 2018. The "Journal of Periodontology," with a global impact factor of 2.768 and a ranking of second, is the publication of choice for Indian periodontists. Journal of Clinical Periodontology, which has an impact Factor of 4.164, is ranked fifteenth in India, but it is the third choice of periodontists worldwide. The top twenty source journals' average impact factor on a worldwide scale is 2.8473. Two journals are placed second on the list of sources at the Indian level.
Jalal et al., 2022 [22]	Bibliometric assessment	Through an analysis of publications in dental science or dentistry in India from 2010 to 2019, the paper concentrated on the expansion and development of this literature. Scopus was used to download 12,830 research papers and 57,793 citations with an h-index of 55. Finding influential authors and influential journals in the field was the study's main goal.	According to the study's findings, the top twenty reputable journals for dentistry science published roughly 64% of the articles written by Indian dental scientists. Journal of Indian Society of Periodontology (6.96%), Journal of Maxillofacial and Oral Surgery (6.33%), and Indian Journal of Dental Research were a few among them. The College of Dental Sciences' Department of Periodontics in Karnataka produced the most publications (n=24).
Kaur et al., 2010 [23]	Bibliometric assessment	The study analyzed India's performance based on its publication output in dental sciences from 1999 to 2008 using a number of parameters, including the nation's average annual growth rate, global publication share and ranking among the world's 25 most productive nations, national publication output and impact measured by the average number of citations per paper, and output and share of international collaborations.	The Journal of Periodontology ranked as the 9^th^ most productive journal publishing Indian papers in dental sciences, with three articles being published between the years 1999-2003 and 26 being published from 2004-2008, a dramatic increase of nearly nine times recorded between the two time periods observed.
Kumar et al., 2021 [24]	Retrospective observational	The purpose of this study was to evaluate the scientometrics of three periodontal journals with high impact factors, identify the contribution of India to these most productive journals during a three-year period (Jan 2018–Dec 2020), and learn about the most significant research subjects. For the Journal of Clinical Periodontology, Journal of Periodontology, and Journal of Periodontal Research, a retrospective observational study was carried out. Every issue of 2018 through 2020 was manually and electronically searched for multiple factors.	Journal of Periodontology published the most articles (469), followed by Journal of Clinical Periodontology (454), and Journal of Periodontal Research (287). China contributed the most to each of the three periodicals, followed by the USA. The majority of articles in the Journal of Periodontal Research have been published by authors from India. Despite being less than their western counterparts, India's publications show a growing trend when examined.
Tarulatha et al., 2021 [25]	Systematic review	In order to statistically analyze the literature on oral hygiene and its relationship to various periodontal conditions in central India, the current bibliometric study was undertaken.	Journal of Periodontology published the second-highest number of articles (26) out of the 1148 shortlisted articles because dental hygiene was thought to be directly related to the prevalence of periodontitis.
Verma et al., 2015 [26]	Bibliometric assessment	The majority of dental professionals in India feel a pressing need to increase their publications due to competition and academic benefits. Therefore, the authors wanted to use a questionnaire to find out how faculty and students felt about scientific publications.	With respect to the subject of Periodontology, 22 articles were published by post-graduate students, senior lecturers published seven studies, readers published 6 and 10 investigations were undertaken by professors, making a total of 45 articles which represented 6.19% of the total 726 studies that were observed in the study.

**Figure 2:**
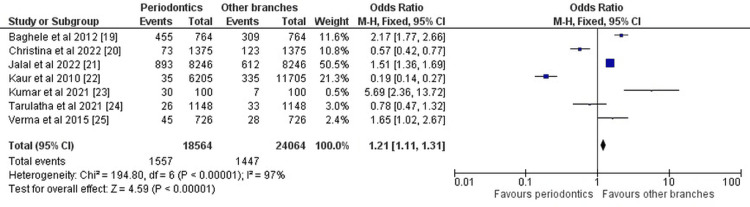
Forest plot for the odds ratio of the studies included Baghele et al., 2012 [20], Christina et al., 2022 [21], Jalal et al., 2022 [22], Kaur et al., 2010 [23], Kumar et al., 2021 [24], Tarulatha et al., 2021 [25], and Verma et al., 2015 [26]

**Figure 3:**
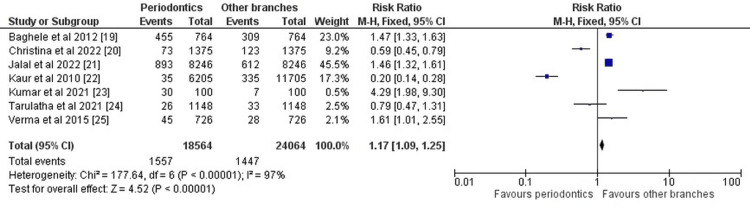
Forest plot for the risk ratio of the studies included Baghele et al., 2012 [20], Christina et al., 2022 [21], Jalal et al., 2022 [22], Kaur et al., 2010 [23], Kumar et al., 2021 [24], Tarulatha et al., 2021 [25], Verma et al., 2015 [26]

Data on the variables analyzed and different aspects of the investigations selected for our systematic review were entered into the RevMan 5 software, and the forest plots representing the risk ratio, and odds ratio were obtained as part of the meta-analysis for our review. Figure 2 and Figure 3 given below show the results of the meta-analysis (using RevMan 5 software) in the form of a forest plot depicting all the studies taken up in this systematic review and evaluating them.

Discussion

The full scope of scientific production is not covered by publication analysis, which was chosen as one way to evaluate scientific advancement. In spite of this, bibliometrics has evolved into a factual aid tool that can plan and create a wide range of data and information handling and board markers, especially in logical, mechanical, and efficiency-related data and correspondence frameworks necessary for organizing, assessing, and managing a specific academic locality or country [12]. This review relied on articles found in the PubMed database; nonetheless, this selection may have underrepresented Indian periodontists' overall impact on the field. Nevertheless, comparable methods have been employed with great success in other partial evaluations of dental research output, and bibliometric studies are more for the distribution of information than for the evaluation of research quality. A piece of writing's reference count should be viewed as a measure of its standing or influence within the scientific community [27]. Due to the complexity and specialization of modern research, it is thought to be impossible for a single researcher to possess all the necessary knowledge and technical abilities. As a result of the complementary nature of the many abilities used in collaboration, new ideas and creativity are stimulated, as well as knowledge exchange. Therefore, collaborative projects not only allow for the efficient pooling and sharing of resources but also improve the quality of the study [28]. Our study had an extraordinarily high degree of author collaboration, with a value of 0.98 (maximum 1).

Comparable goals were pursued by Baghele et al., who used a bibliometric analysis of the PubMed database up to March 1, 2012, to look into the contribution of Indian periodontists to global literature [20]. They observed that the total number of results achieved with the 10 keywords selected for the study was 12,87,077, while only 20,567 hits were produced with the same 10 keywords plus India. According to our keyword research, India contributes an average of 1.45% for each keyword category to the PubMed database. In their study, 764 articles totaling 2,432 authors, 3.2 authors on average per article were published. Only 8.38% of articles were published by lone authors, and the majority of articles had three co-authors. Also, out of 107 journal titles that they had evaluated, 29 belonged to Indian societies and were published there, two were being published there but belonged to international societies, and the remaining 77 were international. There are 181 articles altogether in 41 journals with impact factors of 1.1 or higher. The journal Diabetes had a single article, and the highest IF calculated was 8.889. This study indicates quite clearly the growth of periodontal research in India in terms of both the quantity and the quality of articles that have been presented to world literature, and that too in significantly high IF journals/publications.

While the IF is often used as a quality indicator for academic articles, it should be noted that this metric falls short of being perfect; yet it is the best available and has the added benefit of being tried and true [29]. When it comes to the overall value of diaries within a certain field of study, there are places where both impact factors and diaries shine [30]. As per these data, Indian periodontists dispersed 46.74% of their distributions in diaries with high-impact factors, which is absolutely outstanding. Countless articles were published in journals like the Journal of the Indian Society of Periodontology (JISP), the Journal of the Indian Dental Association (JIDA), the Indian Journal of Dental Research (IJDR), the Journal of Periodontology (JP), the Journal of Contemporary Dental Practice (JCDP), Core Global (QI), the Journal of Periodontal Research (JPR), the Journal of Contemporary Clinical Dentistry (JCD), the Journal of the Academy of Periodontology (JOS), and many more. Despite Arseculeratne's assertion that South Asian articles do not appear as frequently as they should in western journals considered to have high IFs, this is an impressive achievement, especially when the reason is not generally due to low quality, specific ordering, or particular alluding to build IFs [31]. This new direction in science was also disrupted by the virtual absence of logical linkages and stations for propagating research findings in pre-current south Asia. We keep trying for the stars, but there is no equitable way to tally up the money, time, knowledge, and equipment that each researcher, organization, and nation puts into a single research article and one basic test [32].

Since there is a real dearth of independent research being done in India, most of the publications included in this analysis are from academic institutions. The quantity of books and journals has grown dramatically since 2010. Possible causes for this trend include a rise in the total number of dental schools in the country, a rise in the total number of postgraduate seats, the Dental Council of India's policy of "publish and get recognition," a rise in the number of periodontists, a shift in academic policy, more opportunities, a rise in the total number of journals published in India that are indexed in PubMed, and so on. 

A combination of all or some of these variables, or more financing from the public and private sectors is necessary. The maximum number of publications in 2021 was 604, and if these respectable numbers continue, there may be a major shift in the way that Indian periodontists are perceived around the world. The study's findings and analyses may be used by a variety of groups to inform important policies for the advancement of periodontology and dental implantology, including professional societies, individual scientists, academic institutions, and funding organizations.

## Conclusions

Even though we used PubMed as a source of articles, it is possible that it is not representative of the full scope of scientific publishing output and research activity around the world because it is biased toward journals written in English. Still, it is reasonable to assume that the worldwide diary distributions remembered for these data sets accurately address universally acknowledged (or "standard") research, especially the great lab-based essential exploration in the innate sciences, medicine, and life sciences that is led in the profoundly industrialized progressed nations. The Indian literature, which until the 2010s could not be said to be of superior worth and on pace with their western equivalents, has benefited immensely from the contributions of the Indian periodontal community and has increased in the last 10 years in terms of both quality and quantity. Innovating, exploring uncharted territory, developing novel ideas, attending to pressing problems and disputes, and doing fundamental scientific research on the subject of periodontology are all on the rise in our nation right now. While most of the work in the past relied on reiterating the findings of western academics or doing proof-of-concept research, the huge and sustained increase in the number of publications by Indian writers in reputable journals over the past decade is cause for optimism. Given the large number of illustrious periodontists who have contributed to the development, expansion, dissemination, investigation, development, teaching, and mastery of the intricacies of the field of periodontology, it would be grossly unfair to single out a select few for special recognition. The researchers in this study sought to learn more about the research conducted by Indian periodontists by looking through the PubMed database. We did not deviate from this objective in order to assess the specific contributions of Indian periodontists to other databases or sectors, or their abilities in research, clinical practice, or teaching.

Analysis of publishing patterns may serve as a reference for people or government policymakers, administrators, and dental organizations as they formulate future policies and develop programs to enhance scientific and technical knowledge in the area of dentistry. Other nations, unlike India, rely on funding organizations to carry out research programs. Institutions must demonstrate their capabilities and accomplishments in relation to research and publishing in order to do this.
